# The Tri-Lift suspension technique: a modified deep-plane lip lift for enhanced aesthetic outcomes—my personal approach

**DOI:** 10.1186/s40902-025-00459-8

**Published:** 2025-02-08

**Authors:** Bayad Jaza Mahmood

**Affiliations:** Department of Oral and Maxillofacial Surgery, College of Dentistry, University of Sulaimani, Sulaymaniyah, Iraq

## Abstract

**Background:**

The subnasal lip lift has emerged as a popular intervention for correcting an elongated upper lip, although postoperative scarring remains a topic of concern. Various techniques have been discussed in the literature, with the Tri-Lift suspension technique highlighted in this study offering to reduce such complications and to assess the impact of modification in the deep plane subnasal lip lift, which includes triple suspension sutures, on nasal and labial aesthetic parameters.

**Results:**

A total of 193 female patients (mean age: 28 years) underwent the Tri-Lift suspension technique, while 50 female patients (mean age: 32.48 years) underwent the traditional lip lift (bullhorn technique). In the Tri-Lift group, 78.7% reported “very much improvement,” 20.2% “much improvement,” and 1% “no change,” compared to 43% “very much improvement,” 28.6% “much improvement,” 21.4% “improved,” and 7% “no change” in the traditional lip lift group. Quantitative measurements of philtral length, vermilion height, and dental show recorded preoperatively and 6 months postoperatively showed significant improvements in both groups. However, the Tri-Lift suspension technique achieved higher satisfaction rates, fewer adverse outcomes, and statistically significant differences in satisfaction levels (*P* < 0.05) compared with the traditional lip lift.

**Conclusions:**

The Tri-Lift suspension technique offers a solution to the common issue of scarring in subnasal lip lifts, enhancing both nasal and lip aesthetics. It presents a promising alternative to the traditional method, providing higher patient satisfaction and better aesthetic outcomes with reduced scarring.

**Graphical abstract:**

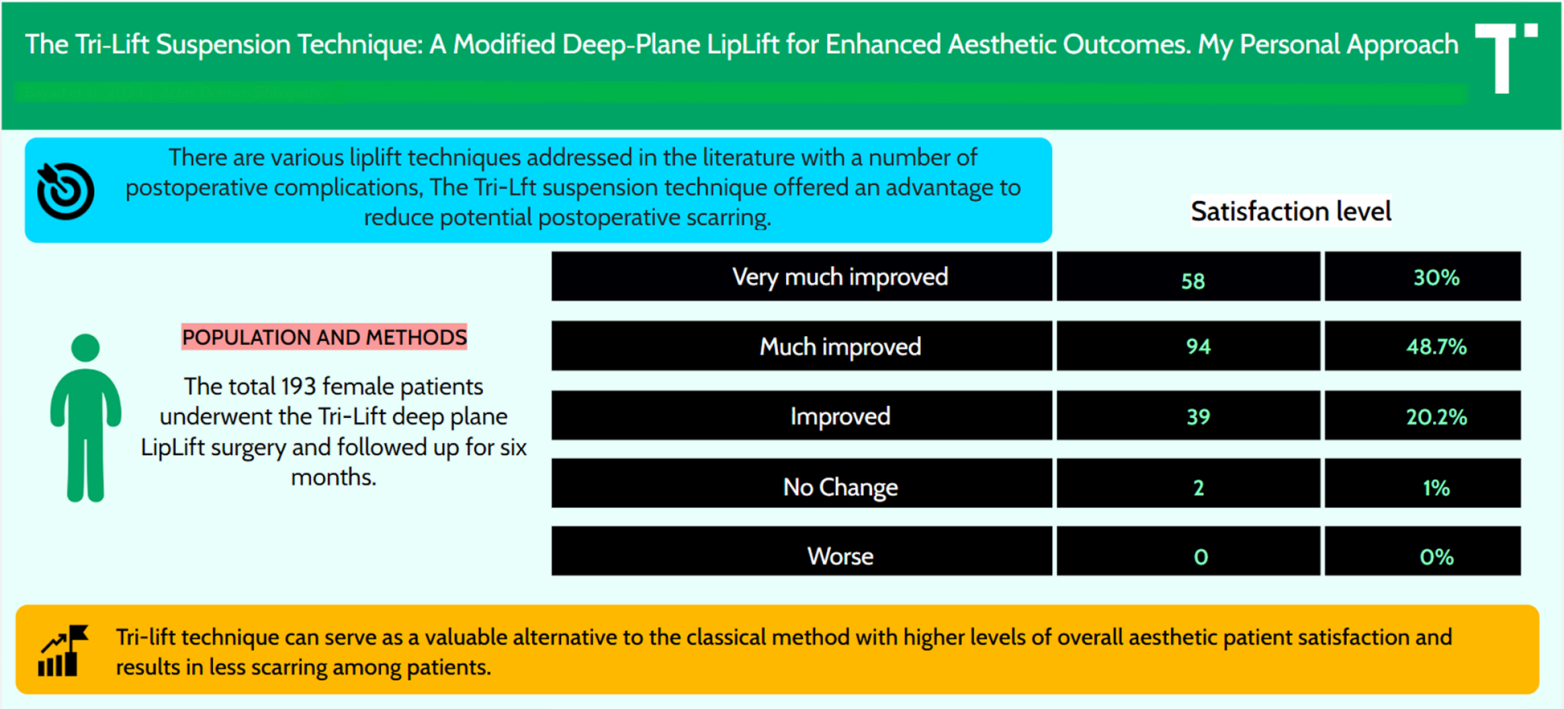

## Background

The perioral region significantly influences facial attractiveness and reflects an individual’s age. As people age, the upper lip undergoes changes such as reduced volume, increased vertical length, disappearance of the cupid’s bow, diminished dental visibility, and loss of lip definition. These alterations, including the straightening of the philtrum and thinning of the vermilion, contribute to changes in facial aesthetics [1–4].

Additionally, these aging characteristics may manifest in the lips of younger individuals due to hereditary factors, prompting them to consider interventions to alter their appearance [5].

Many references discussed the anatomy and function related to aging and the lengthening of the upper lip [6, 7]. The upper cutaneous lip, known as the ergotrid, is a trapezoidal area delineated by the lower boundary of the upper vermilion, the upper boundary of the nasal base, and the bilateral nasolabial folds. Over time, gravity contributes to the descent of the upper lip, leading to increased vertical dimension, flattened philtrum, inverted Cupid’s bow, vermilion thinning, and partial tooth coverage [8].

In the centrofacial area, the upper lip is the only anatomical landmark prone to sagging, unlike other landmarks that remain relatively stable [9, 10]. Therefore, reducing the distance from the nose to the vermilion border of the upper lip is vital for restoring a pleasant and youthful appearance. The effects of a lip lift procedure may be most significant in younger patients, especially those around 35 years old [11].

Various techniques are mentioned in the literature about upper lip lifting procedures, including non-surgical options like filler injections and surgical methods such as simple subnasal skin excision, sub-nostril skin excision, or vermilion advancement [12–15]. The use of filler materials to restore lip architecture and address volume loss in the 1990s [16, 17], while surgical interventions targeting upper lip lift began in the early 1970s, gaining attention with reported cases a decade later [18, 19]. Subsequent developments have concentrated on various excision techniques and methods aimed at reducing scars [20].

Major facial rejuvenation surgeries are common among elderly patients, and the lip lift procedure is underutilized by many surgeons, possibly due to concerns about scarring. However, there is limited research in the existing literature on the impact of lip lift surgeries utilizing dermal suspension on patient satisfaction and scar formation [21, 22].

This study presents a modified technique that improves upon the traditional bullhorn approach with deeper suspension, a centrally vectored advancement flap to reduce tension, and three deep sutures for secure fixation and minimized scarring.

## Patients and methods

A cross-sectional study was conducted to evaluate the outcomes of the Tri-Lift suspension technique, a modified deep-plane subnasal lip lift designed to optimize aesthetic results through enhanced suspension and deeper dissection. This technique was applied to 193 patients (mean age: 28 years), incorporating triple suspension sutures for secure fixation and minimized postoperative complications. As a comparison, the traditional lip lift (bullhorn technique) was performed on 50 female patients (mean age: 32.48 years) to serve as a control group.

The study was conducted between September 2022 and June 2024 at the Dermodento Cosmetic Center, Sulaymaniyah, Iraq. Patients were selected using convenience sampling. Inclusion criteria consisted of medically healthy individuals with no history of upper lip surgeries, permanent lip fillers, or keloid formation. Written informed consent was obtained from all participants. Data collection included patient demographics, complications, and pre- and postoperative measurements of philtral length, vermilion height, and dental show.

Outcome measures included patient-reported satisfaction, assessed at 6 months postoperatively using the global improvement scale and the WHO Quality of Life Questionnaire. Satisfaction was categorized into four levels:

- Very much improved: Significant aesthetic enhancements, with optimal improvements in philtral length, vermilion height, and dental show; no notable complications.
- Much improved: Substantial improvements, though less dramatic, are often influenced by anatomical variations or pre-existing conditions.
- Improved: Noticeable but subtler changes, potentially due to minor concerns or residual asymmetry.
- No change: Minimal impact, likely related to high baseline aesthetics or unmet expectations unrelated to surgery.

Statistical analysis was performed using chi-square tests to compare satisfaction levels and objective outcomes between the two techniques.

The study adhered to the Declaration of Helsinki ethical principles and received institutional approval from the College of Dentistry/Oral and Maxillofacial Department. Quantitative results were presented in a comparative table, demonstrating significantly higher satisfaction rates and fewer complications with the Tri-Lift suspension technique compared to the traditional lip lift (*P* < 0.05).

### Patient selection

Lip lift procedures are suitable for individuals with a noticeable distance between the base of the nose and the upper lip vermilion border, regardless of lip fullness. While many seek lip lifts for rejuvenation purposes, some target congenitally long philtra in younger patients. Candidates are identified during preoperative consultations or through photographic reviews.

A novel classification system has been introduced to identify individuals who are appropriate candidates for lip lift surgery (Table 1) [23]. This system classifies patients based on their labial and philtral height and it is supported by two diagnostic tools: (1) A philtral-labial score (PLS), which measures the ratio of philtral height to upper lip height at the midline, and (2) dental show, which indicates the visibility of the upper incisors when the lips are slightly parted at rest. The utilization of this system helped in identifying suitable candidates for the study.

**Table 1:** The classification system for the upper lip, diagnosis, and surgical management, presuming the absence of maxillary deformities [23]

Type	Philtral height	Labial height	Philtral-labial score	Dental show (mm)	Treatment
0	Normal	Normal	< 3	1–2	None
1	Normal	Short	3–5	≥ 1	Lip augmentation
2	Tall	Normal	3–5	0	Lip lift
3	Tall	Short	> 5	0	Lip augmentation and lip lift

Types 2 and 3 in the classification system feature tall philtra, a philtral-labial score (PLS) of at least 3, and no dental show. These individuals often exhibit upper lip inversion. They are considered ideal candidates for a lip lift surgery if they meet certain health criteria and have normal maxillary height. Corrective measures for skeletal abnormalities should be addressed before soft tissue correction. Another group of candidates includes individuals termed “duckbills,” who have undergone excessive prior lip augmentation, where a lip lift would have been a more beneficial approach [24, 25].

Patients classified as type 0 (defect-free) and type 1 (thin lips) are not ideal candidates for lip lifts. Type 0 patients may consider augmentation, while type 1 lips may show improved vermilion exposure with lifting but risk a shorter philtrum. Additional exclusion criteria include a history of hypertrophic scarring and large ratios of lip width to nasal base or downturned commissures from under correction [13].

Despite the concealing effect of nostril sills in their underlying crease, some surgeons are against indirect lifts for patients with hypoplastic ones [6, 9]. However, in our patient selection process, the presence of prominent sills is not considered necessary, as endonasal scarring eliminates their camouflaging ability. Factors such as age, skin thickness, and Fitzpatrick skin type are regarded as immaterial in our approach [7, 13].

### Surgical marking

**Fig. 1:**
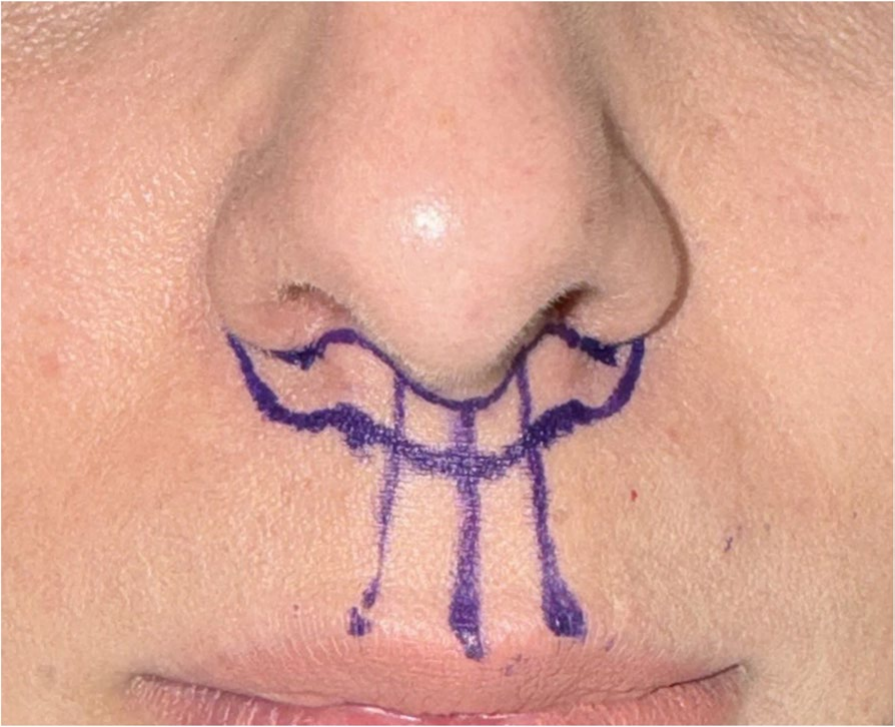
The surgical markings used to define the areas of planned excision for the Tri-Lift suspension technique. The markings ensure symmetrical excision and precise alignment during the procedure (classical bullhorn)

The precise excision outlines and radial reference markings were meticulously drawn on the patient. These markings, based on the classical “bullhorn” lip lift paradigm, are meticulously applied to determine the optimal upper lip excision and ensure symmetrical alignment of the design (Fig. 1).

### Excision

**Fig. 2:**
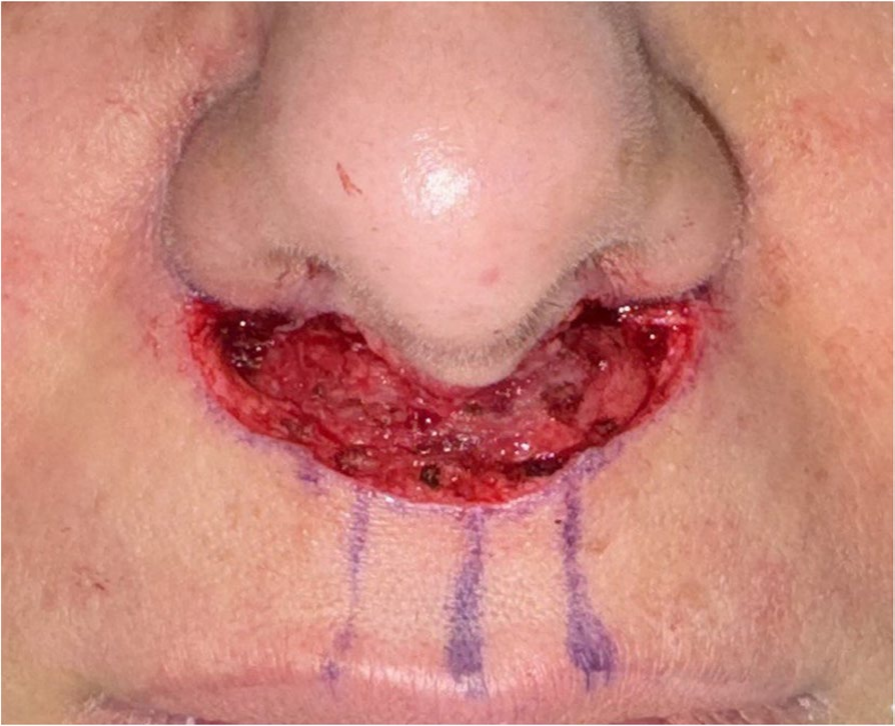
The area after the skin and subcutaneous tissue excision down to the SMAS layer of the upper lip. The excised portion corresponds to the preoperative markings, exposing the underlying musculature for further dissection and suspension

The procedure is performed under local anesthesia with the surgeon positioned at the head of the bed. After prepping the perioral area, the surgeon makes perpendicular lower incisions followed by parallel upper incisions. The skin and subcutaneous flap are then excised over the orbicularis oris muscle, leaving a thin layer of intact fat containing the vasculature (Fig. 2).

### Dissection

After excision, the labial flap is simply elevated in a deep sub-superficial musculoaponeurotic system (SMAS) plane, releasing the labial SMAS from the orbicularis oris muscle below. Centrally extending halfway down to the central philtrum here one can easily feel the hardness of the anterior nasal septum and laterally expose the periosteum at the lateral wall of the nasal aperture (pyriform fossa) for future suture suspension. Care is taken to maintain a sub-SMAS plane to avoid excessive bleeding or damage to the labial elevator complex. Hemostasis is achieved, preferably with bipolar cautery.

#### Suspension and closure

The common mistake among clinicians is relying on basic simple dermal closure methods. Optimal results are achieved by releasing tethering structures and suspending dense tissue upwards. This approach enables the liberated skin/SMAS flap to redistribute tension above the contracted orbicularis oris muscle effectively.

The dermis at the base of the nose lacks firm attachment. Firm suspension points are provided by the periosteum, the pyriform ligament, and the anterior nasal spine. The pyriform ligament, consisting of dense fibrous tissue over the periosteum, spans the pyriform aperture and serves as an optimal site for engaging suspensory sutures [7].

**Fig. 3:**
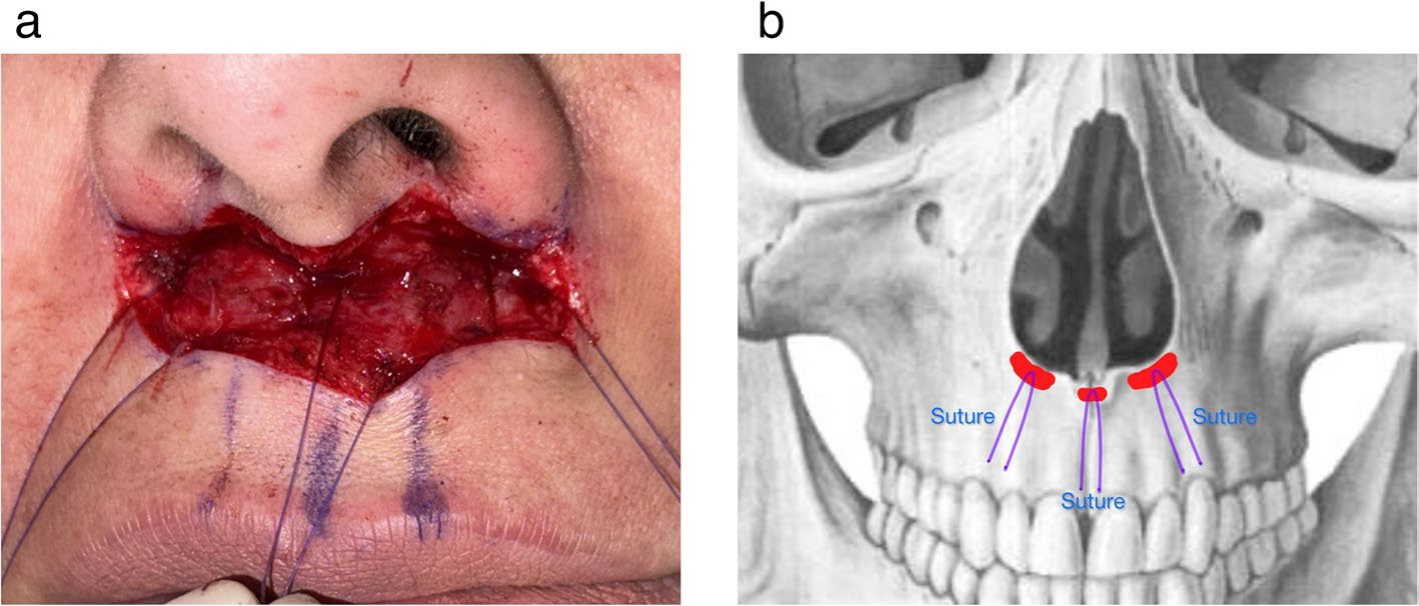
**a** The triple suspension suture on the periosteum of the anterior nasal spine and pyriform aperture. The sutures are secured at key points to elevate the lip and maintain tension for improved aesthetic outcomes. **b** A schematic representation showing the periosteal anchor points for the sutures used during the Tri-Lift suspension technique. The figure highlights the exact anatomical sites for secure and effective lip elevation

The Tri-Lift suspension technique, also known as the triple suspension technique, uses a 5–0 PDS suture, which is resorbable. This is preferred because, after about a month, the labial tissue heals, eliminating the need for non-resorbable materials. The procedure starts by simply passing the needle through the periosteum over the anterior nasal spine at the center, then laterally to the junction of the nasal and oral musculatures. The needle is then positioned deeper to engage the periosteum on the lateral edge of the pyriform aperture, exiting near the alar dermis ensuring not incorporate the dermis (Fig. 3).

Subsequently, the needle is directed inferiorly through the SMAS on the undersurface of the labial flap both centrally and laterally. However, caution must be taken to avoid overtightening the knots, as this may lead to inward enrollment of the labial flap. Suturing to the SMAS rather than the dermis enables the skin to approximate without tension or dimpling. This confers a significant advantage by aligning the dermal edges, given that the SMAS of the upper lip is a distinct tissue layer with considerable strength located just deep to the reticular dermis.

**Fig. 4:**
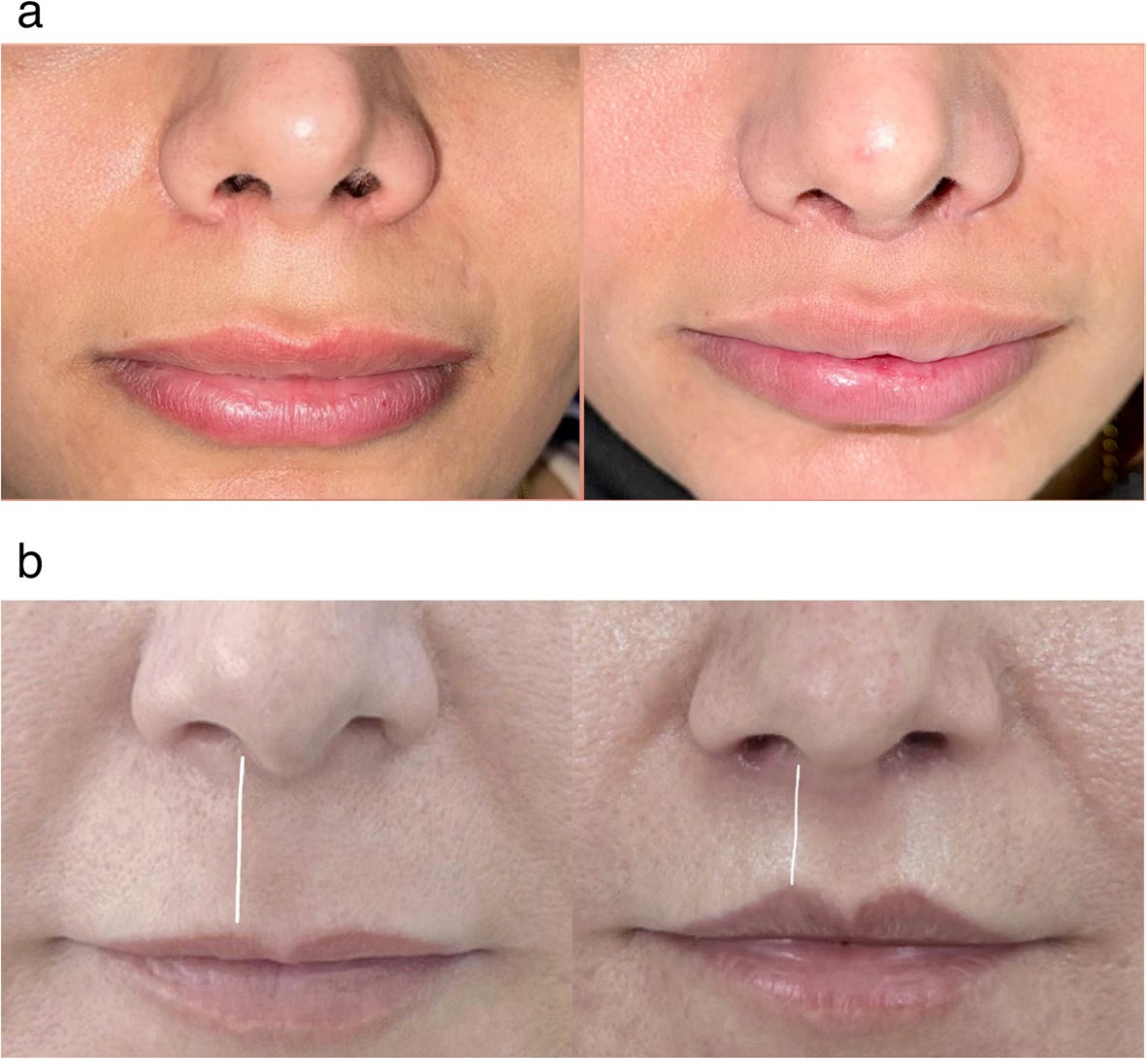
**a** A 6-month follow-up of a 30-year-old woman who underwent the Tri-Lift suspension technique reveals preoperative measurements of a 16-mm philtral length, reduced to 12 mm postoperatively, an increase in vermilion height from 7 to 10 mm, and an improvement in the dental show from 3 to 6 mm. Reported satisfaction level: very much improved. **b** A 6-month postoperative image of a patient treated with the Tri-Lift suspension technique demonstrates a reduction in philtral length from 15 to 10 mm, an increase in vermilion height from 6 to 9 mm, and noticeable improvement in overall aesthetics. Reported satisfaction level: much improved

The incision is subsequently closed further at the subcutaneous level, starting centrally and progressing laterally using a PDS 6.0 suture. This type of suture material is preferred due to its minimal tissue reaction, which can impact healing and scar development. Upon tying the knots, the skin edges should be closely approximated. Finally, reapproximation of the skin surface is achieved through meticulous suturing using 6–0 nylon sutures, ensuring the resolution of any step-offs from the lower to upper skin flaps, and the case is followed up after 6 months for evaluation (Fig. 4a, b).

### Postoperative care

The surgical site should be consistently moistened with antibiotics and anti-scar ointment during the healing period. Patients should be educated about postoperative instructions to prevent disruption of healing and anticipate significant swelling, which may persist for up to 2 months. This prolonged swelling can be attributed to bilateral lymphatic drainage disruption and postoperative myositis induced by muscle trauma.

During the initial 2 months after surgery, stiffness and swelling can aid in incisional healing by limiting movement along the incision line. Patients should attend follow-up appointments every 3 weeks for potential 5-fluorouracil 50 mg/cc injections into firm orbicularis oris patches and may receive fractionated CO_2_ laser treatment for the nasal base if needed, typically administered routinely at 60 and 120 days with a low setting.

## Results

A total of 193 female patients (mean age: 28 years) underwent the Tri-Lift suspension technique, while 50 female patients (mean age: 32.48 years) were treated using the traditional lip lift (bullhorn technique). Statistical analysis using the chi-square test revealed a significant difference in satisfaction levels between the two groups (*P* < 0.05). In the Tri-Lift group, 78.7% of patients reported “very much improved” outcomes, 20.2% reported “much improved,” and 1% reported “improved” or “no change.” In contrast, the traditional lip lift group showed lower satisfaction rates, with 43.0% reporting very much improved, 28.6% much improved, 21.4% improved, and 7.0% no change.

Quantitative measurements, including philtral length, vermilion height, and dental show, were recorded preoperatively and 6 months postoperatively in both groups. The Tri-Lift group demonstrated significant improvements across all parameters, reflecting the effectiveness of the technique.

The comparison of these measurements between the Tri-Lift technique and the control group highlights the means and standard deviations for both, along with statistical analyses such as chi-square values and *p*-values. The data demonstrate that the Tri-Lift technique achieves a significantly greater reduction in philtral length (from 14.50 to 10.75 mm) compared to the control group (from 14.02 to 12.03 mm). Similarly, vermilion height increases more with the Tri-Lift method (from 6.00 to 9.00 mm) than with the control technique (from 5.05 to 7.01 mm), and dental show also improves more substantially (from 2.00 to 5.00 mm versus 1.51 to 3.50 mm). Chi-square tests confirm that these differences are statistically significant, with *p*-values consistently below 0.01. Furthermore, no significant complications, such as infection, bleeding, dehiscence, under-correction, alar distortion, sill widening, or noticeable loss of lift, were observed in either group during the 6-month follow-up period. These findings validate the superior effectiveness of the Tri-Lift technique in delivering improved aesthetic outcomes. The results emphasize the superior outcomes of the Tri-Lift suspension technique in achieving higher satisfaction rates and enhanced aesthetic results compared to the traditional lip lift (Tables 2 and 3).

**Table 2:** Summarization of changes in philtral length, vermilion height, and dental show for Tri-Lift technique (193 cases) and control groups (50 cases)

Measurements	Tri-Lift mean (SD)	Control mean (SD)	Chi-square	*P*-value
Philtral length (pre)	14.50 (1.12)	14.02 (1.00)	9.85	0.0012
Philtral length (post)	10.75 (0.96)	12.03 (1.01)	15.62	0.0004
Vermilion height (pre)	6.00 (0.82)	5.05 (0.51)	11.24	0.0008
Vermilion height (post)	9.00 (0.96)	7.01 (0.49)	18.76	0.0001
Dental show (pre)	2.00 (0.82)	1.51 (0.29)	7.41	0.0065
Dental show (post)	5.00 (0.82)	3.50 (0.32)	13.09	0.0003

**Table 3:** Comparison of satisfaction results between Tri-Lift suspension technique, and traditional lip lift surgery

Patients’ satisfaction with overall aesthetic improvement	Tri-Lift suspension technique (%)	Traditional lip lift surgery (bullhorn) (%)	*P*-values*
Very much improved	78.7	43.0	0.0009
Much improved	20.2	28.6	0.0148
Improved	1.0	21.4	0.0337
No change	1.0	7.0	0.0476

^*^Data analyzed using chi-square tests. Statistical significance was defined as *P* < 0.05

## Discussion

As the global average life expectancy rises and the growing accessibility of aesthetic surgery to address aging, various lip lift techniques have been developed to reduce scarring and improve results. However, much effort has been directed towards modifying incision designs rather than optimizing the lifting process itself [21].

The upper lip lift is a minimally invasive procedure capable of producing a significant facial transformation. By shortening the midface and enhancing dental show and oral visibility, it creates a more youthful and aesthetically pleasing appearance [21, 22].

Between the 1950s and 1980s, the cutaneous upper lip underwent evolutionary changes [19, 20, 26]. During this timeframe, techniques such as the subnasal “bull horn” [27, 28] and supra vermilion “Gull-wing” [29] resections were introduced and gained popularity. However, due to the prominent scarring associated with these methods, some surgeons have opted to discontinue their use altogether [30, 31].

Although many lip lift techniques are effective, comprehensive preoperative counseling about the risk of scarring is essential. Demonstrating the excision area in front of a mirror allows patients to visualize potential results and assess whether their expectations are realistic. The primary drawback of the procedure is the risk of scarring, which may include hypertrophic, atrophic, invaginated, or discolored scars [32, 33].

The Tri-Lift suspension technique differs from other lip plasty methods [18, 19, 21, 22, 34–37] in terms of utilizing a sub-SMAS release with three sutures to stabilize the orbicularis oris muscle, complemented by subcutaneous stitches.

Compared to the other lip lift procedures such as traditional lip lift and dermal suspension flap techniques, the Tri-Lift suspension technique achieved higher satisfaction rates and fewer complications. Traditional methods, like the bullhorn excision, are associated with visible scarring and limited structural support [21], and the dermal suspension flap technique lacks the stability of the triple suture system but may be suitable for thicker skin types [22].

Quantitative measurements, such as philtral length and vermilion height, provide objective validation of surgical outcomes and correlate strongly with patient satisfaction, as demonstrated by similar studies [1, 16]. However, these measurements may not fully reflect patient perceptions, particularly in cases with subtle anatomical changes. Long-term follow-ups are crucial, as tissue remodeling may affect outcomes beyond 6 months [9].

Achieving patient satisfaction following cosmetic procedures is a primary goal for practitioners. In recent years, researchers have extensively studied patient satisfaction levels across various cosmetic treatments, employing diverse self-assessment techniques [22, 38]. This study primarily focused on minimizing tension along the suture line, specifically between the dermal flap beneath the subnasale and the columella. The aim was to improve aesthetic outcomes and achieve the highest level of patient satisfaction.

The key limitation of this study is the sample size, and comparisons with other studies are constrained by variations in patient demographics, surgical methods, and follow-up protocols. Future research should aim to include larger patient cohorts and utilize a variety of satisfaction metrics to offer a more comprehensive understanding of outcomes in cosmetic procedures.

## Conclusion

The Tri-Lift suspension technique has proven to be an effective alternative to the classical excision method, achieving higher overall patient satisfaction with aesthetic outcomes and minimizing scarring.

## Data Availability

The data and materials are available upon request.

## Abbreviations

CO_2_: Carbon dioxide
PDS: Polydioxanone suture
SMAS: Superficial musculoaponeurotic system
WHO: World Health Organization
